# Decoding Encoded Cravings: Epigenetic Drivers of Addiction

**DOI:** 10.3390/brainsci15090927

**Published:** 2025-08-27

**Authors:** Tousif Ahmed Hediyal, Omar Shukri, Elizabeth Stone, Amin Foroughi, Thangavel Samikkannu, Gurudutt Pendyala

**Affiliations:** 1 Department of Anesthesiology, University of Nebraska Medical Center (UNMC), Omaha, NE 68198, USA; thediyal@unmc.edu (T.A.H.); oshukri@unmc.edu (O.S.); elstone@unmc.edu (E.S.); aforoughinezhad@unmc.edu (A.F.); 2 Department of Pharmaceutical Sciences, College of Pharmacy, Texas A&M University, College Station, TX 77843, USA; thangavel@tamu.edu; 3 Department of Genetics, Cellular Biology, and Anatomy, University of Nebraska Medical Center (UNMC), Omaha, NE 68198, USA; 4 Child’s Health Research Institute, University of Nebraska Medical Center (UNMC), Omaha, NE 68198, USA; 5 National Strategic Research Institute, University of Nebraska Medical Center (UNMC), Omaha, NE 68198, USA

**Keywords:** drug abuse, epigenetic modifications, DNA methylation, histone modification, noncoding RNA modifications

## Abstract

Drug abuse is a chronic, relapsing disorder marked by compulsive drug-seeking behavior and profound neurobiological consequences. Each year, millions of individuals face serious social and legal repercussions due to addiction. This review synthesizes findings from both preclinical and clinical studies to examine how chronic exposure to substances such as alcohol, cocaine, methamphetamine, and opioids affects the central nervous system. Specifically, it explores the epigenetic modifications induced by these substances, including DNA methylation, histone modifications, and noncoding RNA regulation. The literature was selected using a thematic approach, emphasizing substance-specific mechanisms and their effects on gene expression, synaptic plasticity, and the brain’s reward circuitry. Emerging evidence links these epigenetic changes to long-term behavioral adaptations and even transgenerational inheritance. This review underscores the complex molecular pathways contributing to addiction, vulnerability, and relapse, offering insights into potential therapeutic targets.

## 1. Introduction

Drug abuse is a chronic relapsing disorder characterized by compulsive drug-seeking behavior and widespread health and social consequences, a dual edged issue with significant public health implications [1]. Each substance of abuse exerts distinct mechanisms of action and toxicities across organ systems, leading to diverse adverse effects [2]. According to the National Survey on Drug Use and Health, 48.7 million American teens and adults (17.3% of the US population) meet criteria for a substance use disorder (SUD). This includes 27.2 million with a drug use disorder (DUD), 29.5 million with an alcohol use disorder (AUD), and 8.0 million with both DUD and AUD [3]. Commonly abused substances such as alcohol, cocaine, cannabis, methamphetamine, and opioids are associated with enduring biological and functional changes that impair physical health, cognitive function and occupational performance.

Chronic drug abuse disturbs the brain’s mesolimbic reward circuitry, which spans the nucleus accumbens (NAc) and the ventral tegmentum (VTA) [4] and interacts with regions like the orbitofrontal cortex (OFC) and limbic system [5,6]. This circuitry plays a central role in reinforcing drug-seeking behaviors, and most addictive substances activate this pathway [7]. Long-term exposure to drugs of abuse leads to persistent alterations in gene expression, protein levels, metabolism, receptor availability, and sensory salience processing. These changes may be substance-specific or shared across drug classes [8]. Traditional neurobiological models, which emphasize acute dopamine signaling and circuit level adaptations, offer valuable insights but fall short in explaining the long-term persistence of craving, relapse risk, and individual variability. Notably, the enduring memory of drug exposure is embedded in transcriptional states [9]. Recent research suggests that integrating epigenetic regulation with circuit based models is essential to fully account for these persistent phenotypes and the heterogeneity observed across substances [10,11].

Susceptibility to drug abuse is shaped by a complex interplay of environmental, social, genetic, and biological factors [12]. Of these, heritable genetic components are known to contribute significantly to vulnerability, accounting for approximately 20 to 50% of the variability in drug abuse risk [13,14]. Chronic exposure induces transcriptional and epigenetic modifications, including DNA methylation, histone modifications, chromatin remodeling, and non-coding RNA regulation. These changes influence reward processing, psychomotor activity, craving, and relapse behaviors [15]. Building on this epigenetic framework, this review has three primary aims: (i) To synthesize current preclinical and clinical evidence on epigenetic mechanisms across major drug classes, identifying both convergent and divergent patterns; (ii) To map existing knowledge gaps that hinder causal inference and cross-substance comparisons, highlighting methodological limitations; and (iii) to evaluate translational opportunities by identifying candidate therapeutic targets among epigenetic regulators and chromatin modifying enzymes (HDCAs, HATs) for future intervention development.

## 2. Epigenetics

**Figure 1:**
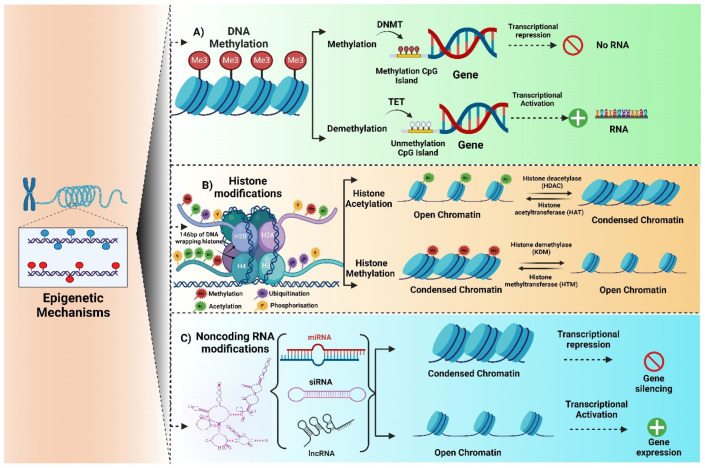
Illustrations of epigenetic mechanisms. (**A**) DNA methylation is an addition or deletion of a methyl group to chromatin structure, which tends to activate or suppress transcription using DNA methyltransferase (DNMT) or ten-eleven translocation (TET) enzymes, respectively, and ends up with or without the production of RNA. (**B**) Histone modification represents several post-translational modifications, including histone acetylation and histone methylation. (**C**) Non-coding RNAs such as miRNA, siRNA, and lncRNA are mainly involved in epigenetic modifications that are responsible for activating and suppressing transcriptional factors.

Epigenetics refers to the regulation of gene expression without altering the underlying DNA sequence [16] (Figure 1). The genome is a complex structure composed of DNA and associated proteins that form chromatin [17]. Epigenetic modifications influence gene expression and subsequent protein synthesis by altering chromatin structure and modulating the accessibility of genes to transcription factors [18]. These changes are heritable and can be shaped by external factors such as environmental exposure and nutrition. Detecting epigenetic modifications provides valuable insights into the mechanisms underlying various physiological and psychological disorders [19]. Three major epigenetic mechanisms are discussed in this review: (i) DNA methylation, (ii) histone modifications, and (iii) three non-coding RNA (ncRNA) mediated gene silencing [20] (Figure 1A–C).

### 2.1. DNA Methylation

DNA methylation is the addition of a methyl group to the regulatory regions, including cytosine, at the 5-methylcytosine (5-mC) and 4-methylcytosine (4-mC) positions and adenine at N6-methyladenine (6-mA) position by DNA methyltransferase enzymes (DNMTs). It is a thoroughly understood epigenetic mechanism in gene regulatory regions [21]. In particular, 5-mC in CpG dinucleotide islands in the genome is the most well-studied mechanism of DNA methylation [22,23]. DNA can either be hypomethylated or hypermethylated in the regulatory regions of specific genes. Hypermethylation, typically mediated by DNMTs, is associated with a closed chromatin structure and reduced gene expression. In contrast, hypomethylation correlates with an open chromatin configuration, promoting transcriptional activation [20]. These methylation states are influenced by developmental signals and external factors such as drug exposure, psychological stress, and disease [24]. DNA demethylation is more complex, mediated by ten-eleven translocation (TET) enzymes. These enzymes remove methyl groups through oxidation, enabling dynamic regulation of gene expression at specific genomic loci [25]. Alterations in DNA methylation patterns play critical roles in biological processes such as transcriptional repression, genomic imprinting, X-chromosome inactivation, silencing of transposable elements, and cell differentiation [23]. By modulating gene expression, DNA methylation contributes significantly to the pathophysiology of various diseases, including substance use disorders [25,26]. Aberrant methylation patterns may serve as early biomarkers for disease diagnosis. Among available techniques, bisulfite treatment remains the most widely used method for detecting and identifying DNA methylation at specific gene loci [27] (Figure 1A).

### 2.2. Histone Modification

Histones are basic proteins that play a crucial role in gene regulation as they organize and regulate DNA into nucleosomes, the fundamental units of chromatin [28,29]. Nucleosomes comprise ~147 base pairs wrapped around the proteins with different subtypes (H2A, H2B, H3, H4). These proteins assemble into an octamer structure composed of two copies and can undergo numerous post-translational modifications (PTMs) such as acetylation, methylation, phosphorylation, ubiquitination, citrullination, SUMOylation, and ADP-ribosylation [30]. Histone modifications lead to structural changes in chromatin, causing vulnerability to transcriptional factors, which contribute to epigenetic changes [31]. They mediate several biological effects on the cell, such as transcriptional activation and inhibition, cell differentiation, DNA replication, and DNA repair and damage [32]. Histone modifications are reported to participate in the epigenetic pathogenesis of several diseases, including drug abuse [32,33,34].

Histone acetylation is among the predominant mechanisms responsible for epigenetic modifications. As the most likely method to unfold chromatin, which is mediated by the histone acetyltransferase (HATs), it reversibly attaches the acetyl group to the lysine residue of histone proteins. Histone can also be deacetylated reversibly by histone deacetylase (HDACs) [31,35], which forms a condensed chromatin structure. Histone acetylation with subsequent chromatin remodeling results in highly affiliated gene expression, whereas histone deacetylation correlates more with silenced gene expression [20]. Studies have found that some histone alterations may affect other epigenetic mechanisms. For example, histone 3 lysine 4 changes (H3K4) could result in decreased DNA methylation by inhibiting the activity of DNMT3 [36].

Histone methylation is also a significant mechanism of histone modification. In this process, the methyl group is transferred from S-adenosylmethionine to amino acids of histone proteins (lysine, arginine, and histidine) by the histone methyltransferase (HMT) enzyme [22]. Methylation of the lysine 4 residue at histone 3 (H3K4) is a well-studied histone methylation site [37]. The histone methylation process is slow and irreversible. Histone demethylases (HDMs), including enzymes such as lysine-specific demethylase 1 (LSD1), tend to remove the methyl group from the histone protein [38]. However, histone methylation is controlled and balanced by adding or deleting the methyl group to the histone protein residues via HMT and HDM enzymes, respectively [39]. The effects of histone methylation on gene expression depend on the targeted histone, the locus of the residue, and the degree of methylation. For example, trimethylation of H3K4 is generally associated with more promoter activation and subsequent gene expression, while trimethylation of H3K27 is correlated with inactivated chromatin and decreased gene expression [31,37]. However, there is an intervening effect of histone methylation between H3K4 and H3K27, which synergistically leads to gene inhibition [40]. Histone methylation alters gene expression by changing chromatin structure [41]. It is also reported that the changes in chromatin compensation can lead to more histone methylation. Further, this influences non-coding RNA and DNA methylation, giving evidence of interference between epigenetic mechanisms [37] (Figure 1B).

### 2.3. Non-Coding RNA Modification

Non-coding RNA (ncRNA) is a class of RNA distinguished from other RNA classes by having fewer biological functions. While mRNA encodes proteins and regulates transcription by activating or silencing gene expression, ncRNA has limited coding potential by comparison [42]. RNAs are mainly divided into two different categories based on size: short-chain ncRNAs (such as siRNAs, miRNAs, and piRNAs) and long-chain ncRNAs (lncRNAs) [43]. A growing body of evidence shows that ncRNAs play a significant role in epigenetic modification and can modulate gene and chromosomal expression to regulate cellular development [44,45]. The most well-studied ncRNAs are small interfering ncRNA (siRNA), long ncRNA (lncRNA), and microRNA (miRNA) [20]. NcRNA mediates epigenetic modifications through several mechanisms. For example, siRNA can enhance DNA and histone methylation, which, in turn, can lead to suppressed gene expression [46]. LncRNA also causes gene silencing by binding with special proteins and forming lncRNA–protein complexes that function directly on chromatin. Likewise, the formation of the CTCF–SRN complex (CTCF is a CCCTC-binding factor that participates in chromatin structure regulation [47]; SRN is one of the types of lncRNA) results in increasing the insulating function of the CTCF protein, which leads to increased chromosomal looping and protects the genes from exposure to other regulatory proteins [48] (Figure 1C).

In stimulant models, cocaine reliably induces the miR-212/miR-132 cluster in the dorsal striatum, where miR-212 acts as a homeostatic brake on drug taking by amplifying CREB–TORC signaling and engaging MeCP2/BDNF feedback loops [49,50,51,52]. Beyond this canonical cluster, other microRNAs, including miR-124 and let-7d, shape cocaine reward and learning in conditioned place preference paradigms, consistent with their roles in synaptic plasticity and transcriptional control. Similarly, opioid exposure elicits a distinct profile: miR-339-3p post-transcriptionally represses the μ-opioid receptor (OPRM1) and is itself upregulated by fentanyl, a pattern compatible with negative feedback on MOR signaling and potential modulation of tolerance and withdrawal dynamics. Methamphetamine models highlighted miR-181a, which contributes to neuroadaptations through ER-stress/ERAD pathways and regulation of GABAA α1 subunits; peripheral signatures, such as elevated plasma miR-9-3p and let-7b-3p, have also been described and may serve as minimally invasive readouts of exposure or phenotype [53,54]. In AUD, multiple miRNAs (Like miR-21, miR-335, miR-9, miR-153), along with region- and tissue-specific changes in miR-124/miR-132, are implicated across brain and peripheral organs, aligning with observed effects on neuroinflammation, synaptic function, and organ system pathology [55,56,57].

lncRNAs are dysregulated in individuals with SUD. Notably, NEAT1, NEAT2, MIAT, and MEG3 were found to be upregulated in the midbrain and NAc of heroin or cocaine users, suggesting a role in transcriptional regulation and nuclear architecture remodeling [58,59]. Across various substances, ncRNA programs converge on core plasticity nodes that integrate molecular and circuit adaptations. For example, miR-212/132 interacts with CREB/TORC, MeCP2, and BDNF to constrain cocaine reinforcement; miR-339-3p regulates OPRM1/MOR signaling in opioid exposure; and miR-181a influences GABAA receptor subunits and ER-stress pathways in methamphetamine exposure. Additionally, lncRNAs such as NEAT1/MEG3/MIAT contribute to transcriptional and chromatin remodeling within reward-related brain circuits. Collectively, these findings outline coordinated regulatory axes that may serve as promising biomarkers of addiction risk and therapeutic interventions [49,60].

## 3. Epigenetic Modifications Induced by Drugs Abuse

**Figure 2:**
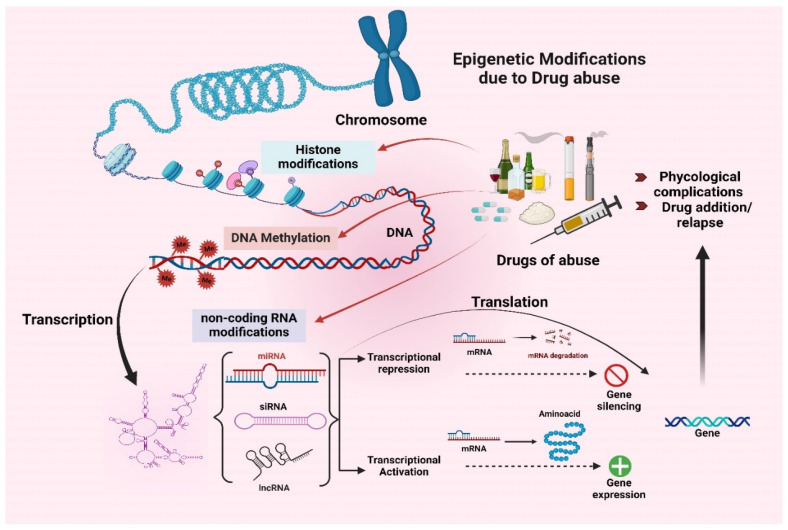
Schematic representation of epigenetic modification due to drug abuse. Exposure to drugs of abuse results in altered epigenetic mechanisms, including histone modification, DNA methylation, and non-coding RNA modifications. Further, it leads to gene silencing or gene activation, which tends to exacerbate psychological complications and drug addiction/relapses.

Drug abuse can lead to addiction, a prevalent issue in our society. The contribution of epigenetic modulation due to drug abuse to the occurrence of and biochemical changes in psychological disorders is not yet fully understood. However, acute or chronic administration of drugs of abuse, including alcohol, cocaine, methamphetamine, and opioids, can lead to significant alterations in gene expression throughout different areas of the brain. Long-term alterations in gene expression in response to chronic drug abuse are steered, at least in part, by epigenetic factors in the absence of DNA sequence variation [61]. Characterizing diverse epigenetic processes and their connection with the global regulation of gene expression profiles may yield insight into the molecular basis of addiction (Figure 2).

Drugs of abuse induce widespread and long-lasting alterations in epigenetic mechanisms, including histone modifications, DNA methylation, and non-coding RNA modifications. These changes play a crucial role in regulating gene transcription, which impacts gene expression and leads to addiction and psychological complications [62,63,64]. Drugs of abuse modify the histone by inhibiting histone deacetylase (HDACs) enzymes. This modification opens the chromatin structure, allowing addiction-related genes to be expressed via synaptic plasticity and dopamine signaling. For instance, histone acetylation at the promoters of the immediate-early genes such as FosB increases their expression by deactivating HDACs, which is responsible for the molecular basis of addiction [65]. Chronic exposure to methamphetamine and alcohol alters histone methylation, with changes in the H3K4, H3K9, and H3K27 regions involved in regulating gene activation and repression. Likewise, suppression of gene encoding proteins involved in synaptic plasticity is controlled by H3K9 trimethylation, whereas H3K4 trimethylation activates genes that are important for long-term behavioral adaptations [66]. Additionally, the phosphorylation of histone H3 at serine 10 (H3S10) has been found to increase in response to cocaine and amphetamines. This post-translational modification increases the chromatin accessibility of the transcription factors to attach to the promoters of addiction-related genes. Indeed, H3S10 phosphorylation is highly correlated with the induction of genes that control dopamine receptor signaling and synaptic strength, which sustain drug-seeking behavior [67]. However, other histone modifications such as ubiquitination and SUMOylation are also responsible for chromatin architecture and transcriptional responses. These modifications play a crucial role in drug addiction. Specifically, histone ubiquitination is implicated in the degradation of chromatin silencing complexes, whereas SUMOylation may act as a counterbalance to promote the formation of silent chromatin configurations [65]. Both mechanisms are involved in modulating dopaminergic signaling and synaptic plasticity, which are key addiction development and maintenance processes. Due to limited data availability, the effect of drug abuse on these mechanisms is still under elucidation.

Chronic usage of substances of abuse like heroin, nicotine, and alcohol increases the CpG methylation of promoters of genes, which are important for synaptic plasticity and the brain’s reward system. For instance, it causes the hypermethylation of genes that encode proteins that are involved in glutamatergic signaling and synaptic remodeling. This reduces their transcription and affects the neural adaptations required for normal reward processing [68]. However, this dysregulation of gene expression strengthens the reinforcing properties of the drugs, leading to addiction. In contrast, hypomethylation of genes involved in addiction, including *FosB* and brain-derived neurotrophic factor (*BDNF*), is observed in individuals with cocaine and methamphetamine addiction [22]. These genes are crucial for long-term neural adaptations, such as structural synaptic changes and enhanced dopamine signaling. Hypomethylation of these genes maintains their chronic activation, sustaining drug cravings and addiction [67]. Importantly, these epigenetic changes are long-lasting even after several months of abstinence and thus lead to an increased risk of relapse. Additionally, chronic use of opioids and cocaine has been associated with heightened DNA hydroxymethylation, with a specific emphasis on higher levels of 5-hydroxymethylcytosine (5hmC) in genes that are linked to synaptic plasticity, stress responses, and reward processing [69]. Addiction-related genes have increased levels of hydroxymethylation in their promoter regions, which increases their expression to bring about behavioral changes in response to prolonged drug use.

MicroRNAs (miRNAs) are also known to be involved in the neuroadaptations associated with drug use. Specifically, MiR-212 is upregulated during cocaine exposure. This miRNA increases cAMP signaling and suppresses compulsive drug-seeking behavior [70]. Both miR-132 and miR-124 regulate genes linked to synaptic plasticity that have been implicated in opioid addiction by influencing neurotransmission and neuronal excitability [69]. Cocaine exposure has been reported to lead to the suppression of dopamine receptor D2 (DRD2) mRNA by siRNA (small interfering RNA), resulting in a decrease in the levels of dopamine receptors in the nucleus accumbens (NAc). This decreased dopamine receptor level alters long-term synaptic plasticity, causing compulsive drug-seeking behavior [67]. This behavior is further strengthened by chronic cocaine use, increasing the levels of lncRNAs such as MALAT1 in the NAc and facilitating chromatin remodeling [71]. Opioid exposure also regulates MALAT1 expression, affecting dopaminergic and glutamatergic signaling [72]. Methamphetamine similarly affects MALAT1 and gene transcription of dopamine receptor signaling.

Nicotine impacts the expression of NEAT1, which may influence neuronal excitability and the long-term reinforcement of smoking behaviors [73]. NEAT1 is upregulated by alcohol, and it affects neuronal plasticity and makes the patient more prone to relapse [74]. Furthermore, MALAT1 and HOTAIR are known to interact with chromatin modifiers and are likely to be involved in alcohol-induced transcriptional regulation of brain reward pathways [75].

While studies have shown that drug abuse impacts miRNA expression and chromatin remodeling in epigenetic modifications, the exact molecular mechanisms that could lead to these changes are not well understood. Future research should focus on identifying the precise pathways through which drugs of abuse alter epigenetic landscapes, transcriptional networks, and neuronal function. A better understanding of these processes could lead to new therapeutic strategies for addiction and substance use disorders.

### 3.1. Alcohol

Alcohol is one of the most consumed psychoactive drugs in the world. Alcohol use disorders (AUD) refer to psychiatric syndromes characterized by impaired control over drinking and other symptoms [76]. AUD is associated with significant epigenetic alterations in the ventral striatum in the human brain. Studies have reported differential methylation regions (DMRs) in the CpG-site of the *SLC30A8* gene, which encodes a zinc efflux transporter important in zinc accumulation in intracellular vesicles in the ventral striatum. Zinc plays a significant role in alcohol breakdown since it is a structure-building element in the alcohol dehydrogenase (ADH) enzyme. This modification may contribute to a change in zinc availability and subsequent impairment of ADH activity in alcohol metabolism [26]. The modulation of *GATA4* gene expression in the orbitofrontal cortex of the human brain is also significantly associated with AUD. The *GATA4* gene encodes the GATA-motif binding protein type 4, a transcription factor that controls the expression of proteins involved in drug metabolism. Epigenetic modification mediates these changes through DNA methylation of the CpG site in the intronic region, leading to chromosomal instability. Furthermore, modulation of *GATA4* gene expression causes epigenetic changes in the DNA hydroxymethylation of CpG sites located in both promoter and intronic regions [77].

A study revealed that chronic alcohol exposure significantly increases epigenetic modification at the whole-genome level in the NAc region of neurons. DNA methylation is mediated by the ability of alcohol to enhance the enzymatic activity of DNA methyltransferase (DNMT) in NAc, which significantly stimulates the gene expression of *Dnmt3a*, and to a lesser extent, the gene expression of *Dnmt1* and *Dnmt3b* at mRNA levels. In addition, it leads to the hypoacetylation of H4 through increasing levels of histone deacetylase (HDAC), which also results in the inhibition of gene expression [78]. Epigenetic modifications such as DNA methylation and histone modification may facilitate dependence-induced neuroadaptations induced by chronic alcohol consumption [79].

It has been reported that alcohol exposure during pregnancy in mice results in epigenetic changes to the offspring’s cells. This includes the demethylation of CpGs sites in the H19 gene in the sperm of the first generation (F1), along with a reduction in spermatogenesis. Additionally, it leads to a decrease in the number of methylated CpGs sites in the H19 gene in the brain of second-generation (F2) offspring [80]. The H19 imprinted gene has been found to regulate the expression of genes responsible for embryonic growth control [81]. Despite these observations, no conclusive relationship between epigenetic modifications and flaws during spermatogenesis has been determined [80].

### 3.2. Cocaine

Cocaine is a tropane alkaloid with a weak base, which is often used as a local anesthetic or sympathomimetic stimulant of the central nervous system. Cocaine abuse is a gradual concern and can lead to drug dependence with time and increased use [82]. Cocaine exposure affects behavior and drug responses in mice through two different mechanisms. First, it controls gene expression by altering the 3D structure of chromatin at specific gene sites in the NAc of the brain. The alteration is in key genes such as *Auts2* and *Caln1* loci, with the implicated mechanisms being decreased CTCF binding (a chromosomal scaffolding protein) and increased *H3K4me3* (trimethylation of Lys4 of histone H3) deposition, and DNA methylation [83]. Second, the translocation of *A2BP1*, a neuron-specific splicing factor that regulates exon inclusion or skipping, into the nuclear region of NAc neurons alters the pre-mRNA splicing of *A2BP1* target genes. Histone modifications mediate these changes, particularly *H3K4me3* in the promoter region [84].

Chronic cocaine exposure leads to long-term dependence through an epigenetic modification in the caudate nucleus of the human brain [64]. This includes hypomethylation of an intragenic region of CpGs in the exon 3 of the Iroquois Homeobox A gene (*IRXA2*), which is a transcriptional suppressor and highly expressed during neural development, and which might be responsible for social behaviors in animals [85]. The cocaine-related DNA methylation of the *IRX2* exon 3 gene leads to alteration in CTCF bindings at the *Auts2-Caln* locus in mice. This results in alterations in 3D chromatin structure and increased *IRXA2* gene expression in the caudate nucleus, which contributes to the development and maintenance of cocaine dependence [85].

Cocaine impacts the central nervous system and results in toxic effects on reproductive functions through multiple epigenetic actions. Consequently, the levels of acylated histones H3 and H4 have been found to be increased in the testes and germ cells after chronic cocaine exposure. A significant decrease in the expression of HDAC 1/2 was also found. On the other hand, DNA methylation levels were found to be elevated in the sperm and germ cells after cocaine treatment, due to alterations in the expression of DNMT 1/2 and the ten-eleven translocation 2 (*Tet2*) gene. The increase in DNMT1 and decrease in DNMT2 and *Tet2* levels are the responsible mechanisms [86].

### 3.3. Methamphetamine (METH)

METH is a psychostimulant of the phenethylamine and amphetamine class of psychoactive drugs, which enhances monoamine neurotransmitters in the nervous system. Currently, the rate of METH dependence is significantly increasing and represents a major burden to the individual as well as to society [87]. Chronic METH exposure leads to dependence and some neuropsychiatric features through significant epigenetic mechanisms. In one study, it was determined that DNA hypomethylation of specified locations (*q*  =  7.04  ×  10^−3^, *p*  =  9.02  ×  10^−9^) and (*q*  =  2.83  ×  10^−2^, *p*  =  1.09  ×  10^−7^) in *CNOT1* and *PUM1* genes, respectively, occurred in blood samples of those exposed to METH. Further, these genes are involved in repressing their target mRNA and promoting mRNA decay and are associated with METH dependence in humans. These epigenetic modifications in mRNA metabolism were found to be like those proposed in both schizophrenia and bipolar disorder [88].

Long-term METH exposure leads to addictive behavior by inducing abnormal methylation of the *CHN2* gene promoter (extracted from peripheral blood samples). This gene encodes for chimeric protein 2 (CNH2), which is associated with the formation of addictive behavior by forming new structures in the actin cytoskeleton of neurons [89]. Moreover, the JAK signaling pathway in the hippocampus (responsible for learning and memory mechanisms) is affected by the abnormal methylation of the *CHN2* gene promoter, resulting in METH addictive behavior [90].

METH abuse also leads to dependence through mediating epigenetic modifications in the glutamate receptors AMPAR and NMDAR promoters in the dorsal striatum region in the brain [62]. These modifications include histone H4 hypoacetylation of the *GluA1* and *GluA2* subunits (promoters of the AMPA receptor) and histone H4 hypoacetylation of the *GluN1* subunit promoter of the NMDA receptor. These changes result in decreased transcription and protein expression of *GluA1*, *GluA2*, and *GluN1*. As a consequence, electrophysiological glutamatergic responses are reduced, ultimately contributing to METH dependence [91].

### 3.4. Opioids

Opium has five naturally occurring alkaloids that are used medicinally: morphine, codeine, thebaine, papaverine, and noscapine. Semisynthetic opioids are derived from one or more of these alkaloids. Heroin (diacetylmorphine) is derived from morphine, as are oxymorphone, dihydromorphone, and hydromorphone. Thebaine (initially called “paramorphine” upon discovery) only makes up 0.1–2.5% of the hydrophenanthrene alkaloids in opium. It cannot be used medicinally due to high toxicity, but instead is a precursor to drugs like oxycodone and hydrocodone. Hydrocodone can also be made from codeine [92].

Heroin is one of the most addictive opioids. Repeated exposure to heroin leads to multiple effects on the body, ranging from the development of seeking behavior to significant physical and psychological impacts on the brain [93]. Egervari et al., 2017 demonstrated in both clinical and pre-clinical studies that the use of heroin is associated with hyperacetylation of lysine residues 27 and 23 of histone 3 (*H3K27ac* and *H3K23ac*) of glutamatergic genes in the Striatum [94]. Therefore, the *H3K27ac* and *H3K23ac* genes promote the opening of the chromatin structure, which increases *GRIA1*’s vulnerability to transcriptional factors, ultimately impairing *GRIA1* function in regulating heroin-related addiction behavior [94]. *GRIA1* is a glutamatergic gene that plays a significant role in regulating synaptic plasticity in the striatum and developing drug-seeking behavior [95,96]. Enhancing glutamatergic transmission in different brain regions is one of the prominent mechanisms involved in the development of rewarding and seeking behavior following exposure to drugs of abuse [97].

Morphine is used as a first-line therapy for relieving both chronic and acute pain, although its clinical application is restricted due to the high risk of addiction development [98]. Long-term exposure to morphine tends to cause glutamatergic transmission in the ventral tegmental area (VTA). Postsynaptic density 95 protein (PSD-95) is a scaffolding protein that enhances glutamatergic synaptic plasticity in multiple brain regions and is encoded by a disk large homolog 4 gene (*Dlg4*). Morphine abuse increases histone 3 (H3) acetylation in the promoter of *Dlg4* by upregulating the expression of phosphorylated cAMP response element-binding (pCREB) protein [99]. CREB is one of the first identified transcriptional factors in mediating morphine addiction [100]. The higher the pCREB activity, the more *PSD-95* expression in the glutamatergic post-synapses. Hence, increased glutamate activity in the synapses of VTA ultimately leads to the development of morphine-induced drug-craving behavior [99]. It has also been indicated that CREB increases the expression of miR-132 in the dental gyrus (DG) [100]. The DG is a cortical region of the hippocampus involved in forming spatial memory and memory of abused drugs [101]. CREB-induced miR-132 overexpression leads to structural plasticity changes in DG neurons by regulating multiple target genes. These changes highlight another aspect of how epigenetic modifications contribute to the development of morphine addiction [100]. Generally, morphine administration is associated with lysine 9 of histone 3 (H3K9) acetylation in the VTA, locus coeruleus, and orbitofrontal cortex. However, chronic morphine administration was found to suppress G9a, a histone methyltransferase that mediates the di-methylation of lysine 9 of H3 (H3K9me2), in the NAc of rat brains. The downregulation of G9a/H3K9me2 is one of the mechanisms underlying the development of morphine addiction-related behaviors [102].

Oxycodone, a μ-opioid receptor agonist, is one of the most addictive prescription opioids [103]. Histone modifications (acetylation and phosphorylation) are mediated by CREB, which is known to be activated by oxycodone [104]. Oxycodone is also known to alter the expression of genes through decreased DNA methylation. As a result, one of the genes affected is the gene that encodes activity-regulated cytoskeleton-associated protein (*Arc*), which is linked to memory, mood, synaptic plasticity, and modulation of the brain’s reward system [104,105]. Another epigenetic effect of oxycodone is the regulation of the transcription of DNA methyltransferases (DNMTs). Downregulation of DNMT1 leads to a series of events that result in physical changes to the hippocampus through increased expression of specific synaptic genes (*SYNAPSIN*, *SHANK2*, and *GAP4*), increased synaptic density, and global hypomethylation [106]. In contrast, studies have shown an increase in DNMT1 in the NAc with heroin use, along with transcriptional upregulation of gamma-aminobutyric acid type A receptor subunit delta (GABRD), which is thought to be accountable for neuroadaptations related to heroin abuse [107].

Both *PSD-95* and Synaptophysin (*Syp*) genes have been found to increase expression in the ventral tegmental area (VTA) following chronic exposure to oxycodone. *PSD-95* and *Syp* are crucial in regulating synaptic plasticity in the VTA. Long-term administration of oxycodone has been linked to hypomethylation of exon 1 and exon 2 of both *PDS-95* and *Syp*. Significant upregulation of *Tet1* and downregulation of DNMTs have been observed in the VTA of rats following chronic exposure to oxycodone. These modifications may be one of the underlying epigenetic modifications in the development of oxycodone-seeking behavior [108].

Prenatal exposure to semisynthetic opioids such as oxycodone can cause memory impairment, particularly in spatial learning and memory [109]. As these children grow, they face increasing struggles. Exposure long before pregnancy, or even exposure to fathers, can cause heritable effects in the next generation. Both types of gamete express opioid receptors [110]. Increased DNA methylation to the *OPRM1* (gene encoding opioid receptor) promoter has been studied in the sperm of opioid (including heroin, prescription opioids, and more) addicts, supporting the possibility of epigenetic effects on the next generation [111].

Increased expression of miR-9 has been shown to increase self-administration of oxycodone [112]. With both oxycodone and hydromorphone use, changes in the regulation of miRNAs have been found after just 24 h of use. Upregulated miRNAs seen in the plasma were let-7a-5p, miR-423-3p, miR-199a-3p, miR-146a-5p, miR-23b-3p, miR-24-3p, miR-221-3p, miR-223-3p and miR-146b-5p. Meanwhile, there were 17 downregulated miRNAs, including miR-144-3p, miR-192-5p, miR-215, miR-363-3p, and miR-194-5p. Hydromorphone caused greater changes in both up- and downregulation than oxycodone did. Some of these miRNAs are thought to be involved in pain signaling and opioid tolerance [113].

Fentanyl is a very potent anesthetic and analgesic opioid. It is widely used to relieve both cancer and noncancer pain by enhancing μ-opioid receptors (MOR). Despite the clinical benefits of fentanyl, it has diverse adverse effects on the central nervous system and pulmonary, cardiovascular, and gastrointestinal systems. Another important side effect is the potential development of dependent behaviors towards fentanyl, as it has rewarding impacts. Chronic treatment with fentanyl decreases drug tolerance and reinforces drug-seeking behaviors, leading to overdose. Fentanyl overdose-related deaths represent a significant health challenge in the meantime [114]. Fentanyl is up to ~100 times more potent than morphine and is more likely to cause addiction [115]. Although there are currently limited studies directly demonstrating that fentanyl induces addiction through epigenetic modifications, its structural and functional similarity to other opioids may offer a plausible explanation. DNA methylation and histone acetylation in brain regions associated with reward and addiction are among the most prominent epigenetic changes observed with substances like morphine and heroin. Given fentanyl’s analogous chemical composition and pharmacological action, it is reasonable to hypothesize that it may exert similar epigenetic effects through comparable mechanisms. Therefore, further preclinical and clinical research is needed to investigate the specific epigenetic impact of fentanyl.

While numerous studies have explored how individual drugs of abuse induce epigenetic changes, there remains a gap in comparing the relative potency of these effects across different substances. Drug pharmacokinetics may offer some insight into this variability. For example, fentanyl is known for its rapid penetration into the brain and its exceptionally high potency, which could result in more pronounced epigenetic modifications compared to other opioids [116]. In contrast, morphine exhibits less favorable pharmacokinetics compared to heroin and oxycodone [117], which may result in less pronounced epigenetic alterations. However, there is a notable lack of information regarding the effects of multiple drugs of abuse on epigenetic modifications. To address this gap, further preclinical and clinical studies are essential to elucidate how various substances influence epigenetic regulation and contribute to addiction-related neurobiological changes.

## 4. Transgenerational Epigenetic Modification Due to Drug Abuse

**Figure 3:**
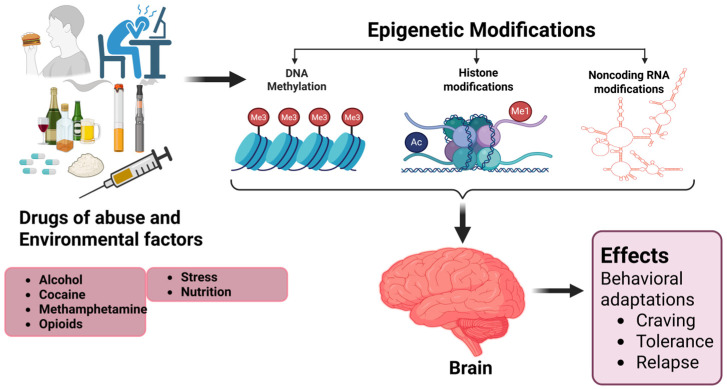
Generalized schematic of epigenetic regulation in the central nervous system (CNS). A schematic overview showing drug of abuse (alcohol, cocaine, methamphetamine, opioids) and environmental factors (stress and nutrition) influence epigenetic mechanisms—DNA methylation, histone modifications, and noncoding RNAs—in key brain regions. These changes affect gene expression and neural plasticity, contributing to behavioral adaptations such as craving tolerance and relapse.

Epigenetic modifications are essential mediators of environmental influences on gene expression without altering the underlying DNA sequence [118]. These mechanisms are susceptible to perturbations during fetal development; thus, drug abuse during pregnancy is a major concern. It has been established that prenatal drug exposure results in alterations in DNA methylation patterns in fetal tissues, which are reflected in long-term changes in gene expression [119]. These changes are especially observed in genes encoding neural development, stress response, and addiction, which suggests possible mechanisms of predisposition to neuropsychiatric disorders. For example, prenatal cocaine exposure relates to reduced DNA methylation at gestational age in newborns, which is a marker of developmental intellectual disability and predisposition to psychiatric diseases in the future [120]. Opioid use during pregnancy has been linked to increased gene expression of *NR3C1*, *OPRM1*, and *BDNF*, which are involved in stress regulation and neurodevelopment [121]. These alterations can be transmitted to the next generation, affecting the sensitivity of offspring to addiction and cognitive dysfunctions [122]. Other investigations have shown that exposure to methamphetamine and cannabis during pregnancy results in DNA methylation alterations at the promoter regions of genes encoding proteins involved in dopaminergic signaling and synaptic plasticity, including *DRD2* (Dopamine Receptor D2) and *SYN1* (Synapsin 1), which in turn may contribute to neurodevelopmental abnormalities [123,124] (Figure 3).

Histone modifications, including acetylation and methylation, are crucial for chromatin architecture and gene expression [125]. Methamphetamine use during pregnancy has been found to lead to histone modifications in the fetal brain, which affects the expression of TH (Tyrosine Hydroxylase), DAT (Dopamine Transporter), and COMT (Catechol-O-Methyltransferase), all of which are increased in individuals predisposed to addiction [123]. In addition, studies conducted on prenatal morphine exposure have reported changes in histone methylation in the placenta, regulating nutrient transport and fetal growth, especially the *H19* and *IGF2* genes [121].

Prenatal cocaine exposure has been connected to histone deacetylation of *BDNF* and *NR3C1*, which downregulates their expression and adverse neurodevelopmental consequences that lead to enhanced sensitivity to stress-related disorders [120]. Similarly, cannabis has been found to influence histone modifications of genes such as *SYN1* and *GRIA2*, which control synaptic signaling and cognitive function and may predispose offspring to memory dysfunction and neuropsychiatric morbidity [124]. Additionally, prenatal exposure to psychostimulants such as amphetamines has been linked with reduced histone acetylation of *FOXP2*, a gene important for language and cognition, which may result in poor performance in verbal tasks and executive functioning in the offspring [126].

Recent studies show that non-coding RNAs, such as microRNAs (miRNAs), are important in fetal development and are easily influenced by maternal drugs. It has been established that cannabis use during pregnancy has adverse effects on the expression of miRNAs in the fetal brain, including miR-125b, miR-132, and miR-29, which are involved in learning and memory processes. Also, prenatal opioid use has been linked to miR-146a, miR-181, and let-7 dysregulation, which are involved in neural differentiation, synaptic plasticity, and immune regulation, respectively [124]. These changes have been found to increase the vulnerability of the offspring to neuropsychiatric disorders and to attenuate their stress responses. In addition, studies on prenatal methamphetamine exposure have established that miR-9 and miR-124 are downregulated, both of which are critical for normal brain development, neuronal differentiation, and synaptic plasticity [127]. The dysregulation of these miRNAs may lead to cognitive impairment and increased susceptibility to addiction in later life. Moreover, miR-34c is known to be regulated by cocaine, and this miRNA is involved in dopamine signaling and neuroprotection. Downregulation of this miRNA leads to increased oxidative stress and neuronal apoptosis, leading to long-term neurodevelopmental consequences [128].

Disruptions in brain development are associated with prenatal drug exposure. Cocaine-exposed infants have been found to have altered expressions of *BDNF, GRIN2B*, and *RELN*, which are very important regulators of neuronal growth and synaptic plasticity [120]. These molecular disruptions are risk factors for cognitive impairment, ADHD, and psychiatric disorders at later ages [129]. The placenta is the first organ to encounter the drugs while regulating the exchange of nutrients and oxygen. Morphine and opioid exposure have been incriminated in causing epigenetic alterations in placental genes that control angiogenesis and the immune system, namely *VEGFA, FLT1*, and *IGF2*, resulting in placental insufficiency and fetal growth restriction [121]. Similarly, methamphetamine has been seen to affect the placental function and increase the risk of preterm birth and low birth weight [123]. Prenatal drug exposure can affect the fetal hypothalamic-pituitary-adrenal axis, which controls the stress responses. Infants of mothers with substance abuse have been found to have epigenetic modifications of *NR3C1* (Glucocorticoid Receptor), *FKBP5*, and *CRH* (Corticotropin-Releasing Hormone), which result in altered stress responses, and these children are at higher risk of developing anxiety and depression in adulthood [129].

In addition to drugs of abuse, various environmental and maternal factors can significantly influence epigenetic modifications during both prenatal and postnatal development [130]. Maternal cigarette smoking, nutrition, and psychosocial stress are among the key contributors, acting individually, synergistically, or antagonistically to alter epigenetic patterns [131]. For instance, in utero exposure to cigarette smoke has been shown to modify DNA methylation in genes critical for fetal brain development, potentially leading to long-term neurological consequences [132]. Similarly, a maternal diet rich in folic acid has been associated with hypomethylation of genes regulating insulin-like growth factor 2, which plays a vital role in fetal growth and development [133]. Moreover, maternal depression and anxiety during pregnancy have been linked to increased internalizing symptoms such as depression and social withdrawal in offspring during childhood and adolescence. This effect is mediated by elevated methylation of the *NR3C1* gene, which encodes the glucocorticoid receptor, resulting in dysregulation of the hypothalamic-pituitary-adrenal (HPA) axis and increased cortisol levels in infants [134]. However, further research is needed to clarify the confounding and interactive effects of multiple environmental factors on epigenetic modifications during prenatal and postnatal periods in order to accurately isolate the impact of individual variables.

## 5. Conclusions

Drug abuse affects millions of people every year, resulting in serious social and legal consequences. Chronic drug use can “rewire” the mesolimbic reward system through epigenetic changes. Alcohol, cocaine, methamphetamine, and opioids are prominent drugs of abuse in our society and are associated with both psychological and epigenetic modifications. Epigenetic pathways, including DNA methylation, histone modifications, and noncoding RNA regulation, play a critical role in mediating the neurobiological effects of drugs of abuse. These mechanisms influence gene expression in key brain regions involved in reward processing, stress response, and cognitive control, thereby contributing to the development and persistence of addictive behaviors [135]. Despite significant advances in understanding these processes, the precise relationship between epigenetic modulation and addictive behavior remains incompletely understood and warrants further investigation.

Considering that epigenetic changes due to substance abuse have been found not only in the abused brain but are also considered to be capable of transgenerational inheritance, it would be worth investigating how many generations of drug abuse-related changes to the brain might persist. A large variety of studies have previously shown that many types of stressors to parents (including starvation, obesity, trauma, and drug use) do cause epigenetic changes in offspring [136]. It would be valuable to determine how many generations are affected by drug-induced epigenetic changes and whether these effects vary across different environments, types of prenatal care, and psychosocial conditions. Rodent models offer a promising approach for such investigations due to their rapid generational turnover and the availability of well-established murine addiction paradigms. These models can help elucidate the transgenerational impact of substance abuse on epigenetic regulation and provide insights into environmental interactions.

Importantly, understanding these epigenetic processes opens new avenues for therapeutic intervention. Reversing maladaptive gene expression patterns may be achievable through emerging approaches such as noncoding RNA-based therapies and histone deacetylase (HDAC) inhibitors. Future research should focus on refining these strategies, enhancing the specificity of delivery to targeted brain regions, and exploring their potential for personalized addiction treatment.

## Data Availability

Not applicable.

## Abbreviations

The following abbreviations are used in this manuscript:

SUD	Substance use disorder
AUD	Alcohol use disorder
NAc	nucleus accumbens
DNA	Deoxyribonucleic acid
miRNA	microRNA
siRNA	small interfering ncRNA
DNMT	DNA methyltransferase
HDACs	Histone deacetylase
ncRNA	Non-coding RNA
RNA	Ribonucleic acid
METH	Methamphetamine
